# Long‐term trends in critical care admissions in Wales* ^*^

**DOI:** 10.1111/anae.15466

**Published:** 2021-05-02

**Authors:** R. J. Pugh, R. Bailey, T. Szakmany, M. Al Sallakh, J. Hollinghurst, A. Akbari, R. Griffiths, C. Battle, C. Thorpe, C. P. Subbe, R. A. Lyons

**Affiliations:** ^1^ Department of Anaesthetics Glan Clwyd Hospital Bodelwyddan UK; ^2^ Public Health Medicine Swansea University Swansea UK; ^3^ Department of Anaesthesia, Intensive Care and Pain Medicine Division of Population Medicine Cardiff University Cardiff UK; ^4^ Public Health Medicine Swansea University Swansea UK; ^5^ Public Health Medicine Swansea University Swansea UK; ^6^ Public Health Medicine Swansea University Swansea UK; ^7^ Public Health Medicine Swansea University Swansea UK; ^8^ Ed Major Critical Care Unit Morriston Hospital Swansea UK; ^9^ Department of Anaesthetics Ysbyty Gwynedd Bangor UK; ^10^ Acute and Critical Care Medicine School of Medical Sciences Bangor University Bangor UK; ^11^ Public Health Medicine Swansea University Swansea UK

**Keywords:** ageing, comorbidity, critical care capacity, frailty, outcomes

## Abstract

As national populations age, demands on critical care services are expected to increase. In many healthcare settings, longitudinal trends indicate rising numbers and proportions of patients admitted to ICU who are older; elsewhere, including some parts of the UK, a decrease has raised concerns with regard to rationing according to age. Our aim was to investigate admission trends in Wales, where critical care capacity has not risen in the last decade. We used the Secure Anonymised Information Linkage Databank to identify and characterise critical care admissions in patients aged ≥ 18 years from 1 January 2008 to 31 December 2017. We categorised 85,629 ICU admissions as youngest (18–64 years), older (65–79 years) and oldest (≥ 80 years). The oldest group accounted for 15% of admissions, the older age group 39% and the youngest group 46%. Relative to the national population, the incidence of admission rates per 10,000 population in the oldest group decreased significantly over the study period from 91.5/10,000 in 2008 to 77.5/10,000 (a relative decrease of 15%), and among the older group from 89.2/10,000 in 2008 to 75.3/10,000 in 2017 (a relative decrease of 16%). We observed significant decreases in admissions with high comorbidity (modified Charlson comorbidity index); increases in the proportion of older patients admitted who were considered ‘fit’ rather than frail (electronic frailty index); and decreases in admissions with a medical diagnosis. In contrast to other healthcare settings, capacity constraints and surgical imperatives appear to have contributed to a relative exclusion of older patients presenting with acute medical illness.

The proportion of people aged ≥ 85 years in the UK is expected to double over the next 25 years in line with global trends [1]. The attendant acute and chronic illnesses in this group represent a significant driver for increased demands on critical care services [2, 3, 4]. However, while an increase in admissions of older patients (> 80 y) to critical care has been observed in Australia [5], some European nations [6, 7, 8], and for areas of the UK (excluding Scotland) [9], these trends are not universal among developed countries [10, 11]. Indeed, the recently observed decrease in older patients admitted in Scotland has raised concerns over rationing of admission according to chronological age and risks of inequitable access [11].

We investigated the potential effects of resource constraints on admission patterns and processes of care in Wales, a UK nation with critical care capacity much lower than the reported European average (5.7 vs. 11.5 per 100,000 population; for comparison with other developed nations, see online Supporting Information Table S1) [12, 13]. The purpose of this study was to investigate trends in patient characteristics for adult critical care admissions between 2008 and 2017. We hypothesised that without an increase in capacity, critical care admission characteristics may not follow national population trends, and that with resource constraints there may be a decreasing tendency to admit those with significant underlying illness. As such, although the project was conceived and conducted before the outbreak of COVID‐19, the themes could be even more relevant given the recent acute stress on resources.

## Methods

We used the Secure Anonymised Information Linkage Databank (www.saildatabank.com) to carry out all analyses. The development of this Databank has been described previously [14, 15, 16]. The project received approval from the independent Information Governance Review Panel, Swansea University.

We identified adult critical care admissions in Wales between 2008 and 2017 from the Critical Care Dataset (collated from the monthly Critical Care Minimum Dataset exports from Welsh ICUs and Patient Episode Database for Wales). We linked this information to the Welsh Demographic Service Dataset, the Welsh Longitudinal General Practice data and the Annual District Death Extract from the Office for National Statistics mortality statistics. We extracted national population trends data from an independent open data source [17]. Changes in critical care capacity over the period were identified from contemporary Welsh Critical Care and Trauma Network reports.

We restricted inclusion to episodes with high‐quality matching from the identity linkage and anonymisation process for individuals who were aged ≥ 18 years on the day of critical care admission and registered to a residential address in Wales. Patients were followed‐up until one year after hospital discharge, death or outward migration, whichever occurred first.

We categorised patients according to age as follows: 18–64 (youngest); 65–79 (older); and ≥ 80 y (oldest). We calculated a modified Charlson comorbidity index on the date of critical care admission using the ICD‐10 codes [18] within the Patient Episode Database for Wales and a look‐back period of one year [19]. We categorised comorbidity according to modified Charlson comorbidity index as: low (‐1–0); medium (1–10); and high (> 10). Frailty was determined using the electronic frailty index (eFI) derived from Welsh Longitudinal General Practice data and recently implemented in Wales in those aged ≥ 65 years [20, 21]. We calculated the eFI according to date of critical care admission using 10 years of previous general practitioner data for each individual and used this score to categorise as: fit (eFI value 0–0.12); mild (> 0.12–0.24); moderate (> 0.24–0.36); or severely frail (> 0.36).

We explored annual trends in admissions for each age cohort and tested for significant changes over the study period. Differences in proportions of patients according to comorbidity index and eFI category were compared across years using Chi‐squared tests for trends. We analysed counts and crude (unadjusted) incidence rates of admissions per 10,000 population using Poisson regression models, with the variable for year of admission added as the independent variable and national population estimates for each age group added as the offset. We converted model coefficients to rate ratios to compare differences across years compared with the baseline year (2008). We used separate models to analyse rates of admissions requiring the following: advanced respiratory support (typically mechanical ventilation); advanced cardiovascular support (multiple vaso‐active/anti‐arrhythmic drugs and/or cardiac output monitoring, intra‐aortic balloon pump or temporary cardiac pacemaker); and renal support (renal replacement therapy). We categorised rates by admission type as medical, surgical or other, and as planned or unplanned. Proportions of admissions with a recorded death were explored (critical care; post‐critical care in‐hospital; and total in‐hospital mortality) and were tested for significant changes over time for within each age group. One‐year mortality was investigated from point of hospital discharge following index critical care admission and again tested for significant changes over time within each group. We considered values of p < 0.05 to be statistically significant.

## Results

**Figure 1:**
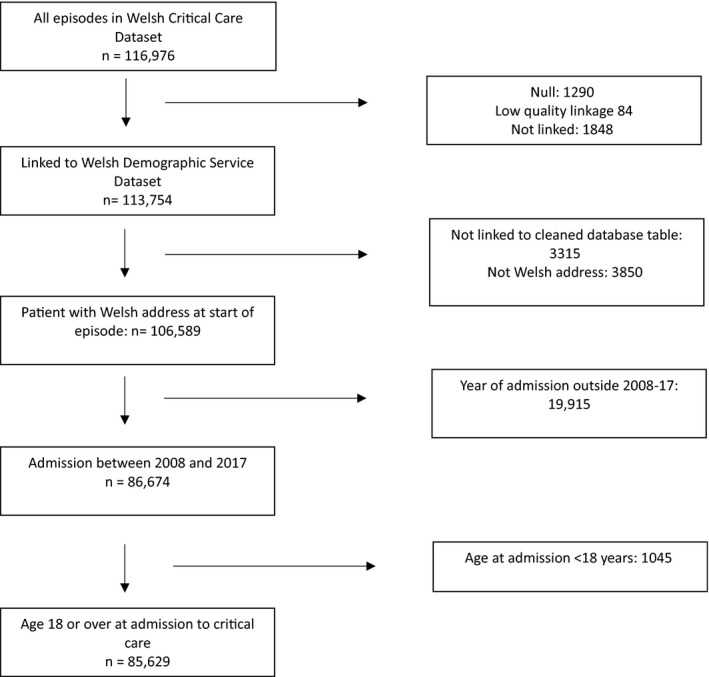
Study diagram.

We identified 85,629 admissions aged ≥ 18 years admitted to critical care units in Wales between 1 January 2008 and 31 December 2017 (Fig. 1). During this time, the number of critical care beds decreased from 178 to 167, a change associated mainly with the closure of small units based within satellite hospitals [12, 22]; however, nine additional post‐anaesthesia care unit (PACU) beds were opened between April and August 2015, all of which contributed to the Critical Care Dataset (online Supporting Information Table S2). Median (IQR [range]) number of annual admissions was 8521 (8349–8913 [7955–9046]) (online Supporting Information Table S3); there were no statistically significant changes during the study period. However, nationally, there was an increase in the resident population aged ≥ 18 years from 2,385,972 in 2008 to 2,496,876 in 2017, with increases particularly among those aged 65–79 and ≥ 80 years (online Supporting Information Table S4). Thus, we observed an overall decrease in the rate of admissions from 37.7/10,000 in 2008 to 35.9 /10,000 in 2017 (χ^2^ = 171.4, p < 0.001; Supplementary Figure S1).

**Figure 2:**
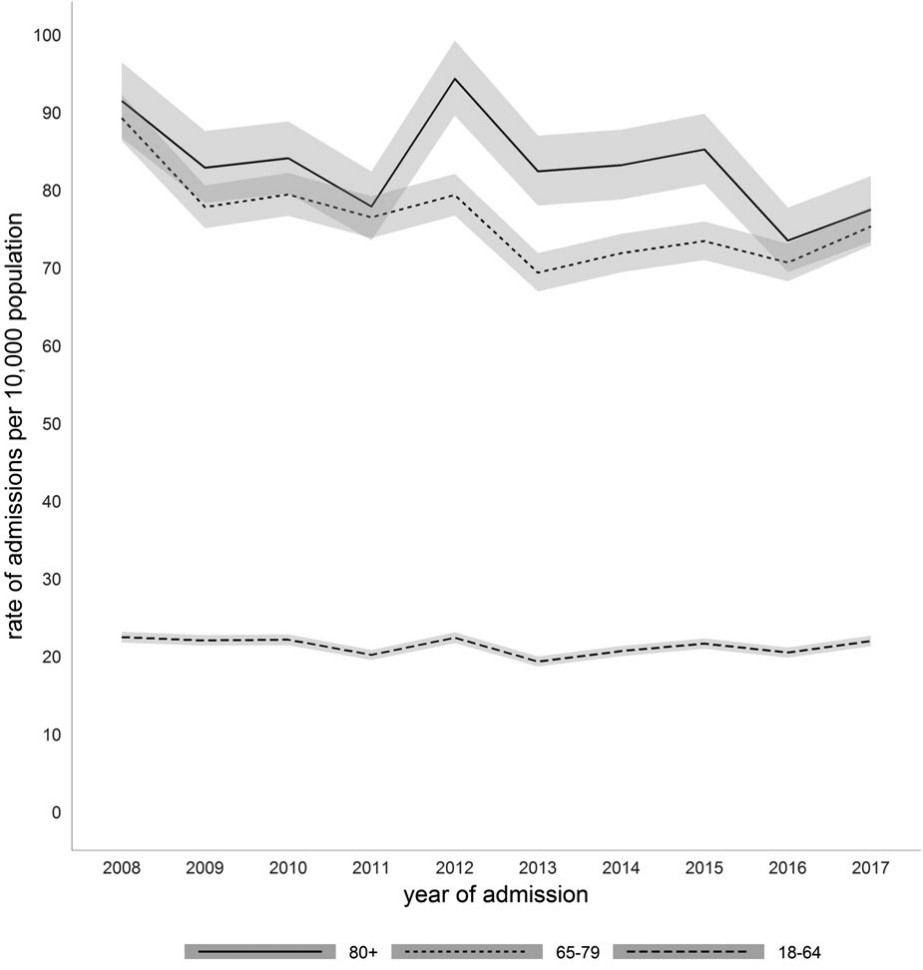
Rates of ICU admission per 10,000 population over time by age group with 95%CIs.

The oldest age group (≥ 80 years) accounted for 15% of admissions to critical care, the older age group (65–79 years) for 39% and the youngest age group (18–64 years) for 46% (Table 1). These proportions did not change significantly over the study period. However, in relation to the national population, the incidence of admission rates to critical care per 10,000 of the oldest group significantly decreased from 91.5/10,000 in 2008 to 77.5/10,000 in 2017 (p < 0.001), representing a relative decrease of 15% (RR 95%CI 0.85 (0.78–0.91)). Similarly, the incidence of critical care admissions in the older (65–79 y) age group fell from 89.2/10,000 in 2008 to 75.3/10,000 in 2017 (p < 0.001), a relative decrease of 16% (RR 95%CI 0.84 (0.81–0.88)). There were no significant changes in admission rates per 10,000 population in the youngest age group (RR 95%CI 0.98 (0.94–1.02), p = 0.34)) (Fig. 2).

**Table 1:** Case‐mix, processes of care and outcomes of ICU admissions according to age group. Values are number (proportion).

**Age cohort**	**18–64 y**	**65–79 y**	**80 y and older**	**p value**
n	39,551 (46.2%)	32,928 (38.5%)	13,150 (15.4%)	
Female	18,638 (47.1%)	14,126 (42.9%)	6508 (49.5%)	< 0.001
Medical	18,417 (46.6%)	12,222 (37.1%)	4341 (33.0%)	< 0.001
Surgical	20,443 (51.7%)	19,984 (60.7%)	8501 (64.6%)	
Other specialty	691 (1.8%)	722 (2.2%)	308 (2.3%)	
Planned admission	8543 (21.6%)	9639 (29.3%)	3250 (24.7%)	< 0.001
Unplanned admission	30,525 (77.2%)	22,925 (69.6%)	9788 (74.4%)	
Low comorbidity	9457 (23.9%)	2056 (6.2%)	772 (5.5%)	< 0.001
Medium comorbidity	10,983 (27.8%)	5921 (18.0%)	1845 (14.0%)	
High comorbidity	19,111 (48.3%)	24,951 (75.8%)	10,583 (80.5%)	
Fit	26,170 (66.2%)^a^	13,930 (42.3%)	4235 (32.2%)	< 0.001
Mild frailty	9944 (25.1%)^a^	11,202 (34.0%)	4110 (31.2%)	
Moderate frailty	3084 (7.8%)^a^	6697 (20.3%)	3925 (29.8%)	
Severe frailty	353 (0.9%)^a^	1099 (3.3%)	880 (6.7%)	
Advanced RS^b^	17,765 (44.9%)	12,551 (38.1%)	4110 (31.2%)	< 0.001
Advanced CVS^c^	4976 (12.6%)	5394 (16.4%)	1935 (14.7%)	< 0.001
Renal replacement	3879 (9.8%)	3868 (11.7%)	1110 (8.4%)	< 0.001
Critical care mortality	4176 (10.5%)	5766 (17.5%)	2692 (20.4%)	< 0.001
Hospital mortality	5466 (13.8%)	8174 (24.8%)	4382 (33.3%)	< 0.001
One‐year mortality	6389 (16.1%)	10,058 (30.5%)	5823 (44.2%)	< 0.001

^a^ Electronic frailty index has not been validated in those aged < 65 y.

^b^ Advanced respiratory support (i.e. invasive mechanical ventilation).

^c^ Advanced cardiovascular support (i.e. multiple vaso‐active/anti‐arrhythmic drugs and/or cardiac output monitoring, intra‐aortic balloon pump or temporary cardiac pacemaker).

A high degree of comorbidity was present in 63.8% of all admissions and was most prevalent in the oldest group (Table 1). During the study period, we observed a significant decreasing trend in the proportion of admissions with high comorbidity across all groups. There was a relative decrease of 6.9% in the oldest age group (χ^2^ = 14.1, p < 0.001), from 79.1% in 2008 to 73.7% in 2017, a decrease of 12.7%; in the older age group (χ^2^ = 195.7, p < 0.001) from 78.7% in 2008 to 71.5% in 2017; and a decrease of 12.2% in the youngest age group (χ^2^ = 52.8, p < 0.001) from 49.7% in 2008 to 43.6% in 2017.

Of all admissions aged > 65 years (both the older and oldest groups), 60.6% were categorised as at least mildly frail, and 27.3% were moderate or severely frail, again highest in the oldest cohort. Although there were no significant trends in the proportions within each frailty category for oldest patients, in the ‘older’ cohort there was a small but statistically significant increase in the proportion of ‘fit’ patients, from 43.4% in 2008 to 45.3% in 2017 (χ^2^ = 4.95, p = 0.03), and a small but significant decrease in the proportions with mild frailty from 35.8% in 2008 to 33.5% in 2017 (χ^2^ = 5.14, p = 0.02). Noting the limitations regarding frailty assessment in individuals aged < 65 y, there was a significant increase in the proportion of younger patients with ‘moderate and severe frailty’, from 6.6% in 2008 to 8.9% in 2017 (χ^2^ = 25.53, p < 0.001); and a decrease in the proportion of ‘fit’ patients among the youngest cohort, from 70.7% in 2008 to 64.6% in 2017 (χ^2^ = 52.79, p < 0.001).

**Figure 3:**
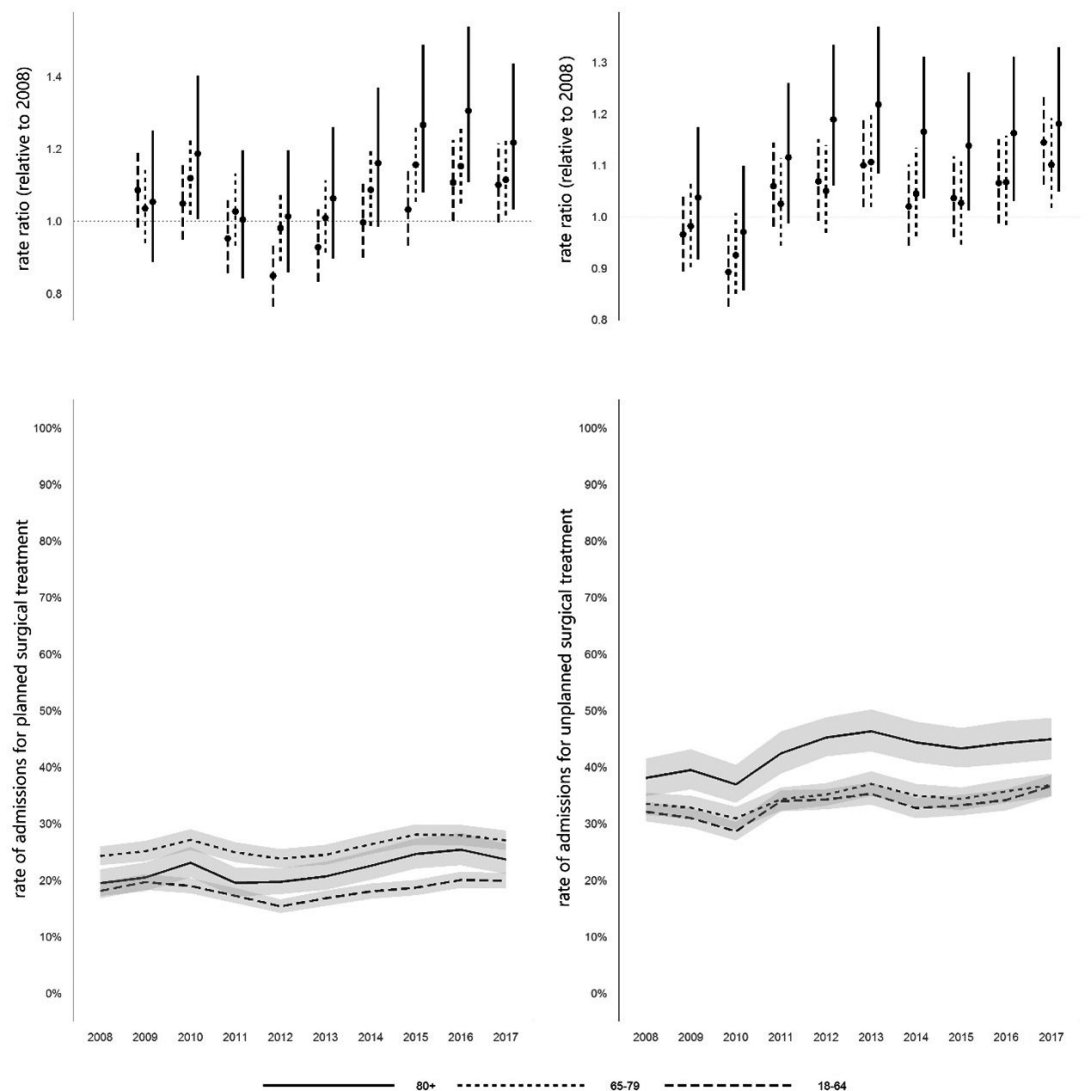
Rates of admission for planned and unplanned surgical treatment 2008–2017. Values are rate ratios relative to 2008, and rates of admission for planned and unplanned surgical intervention, with 95%CIs.

**Figure 4:**
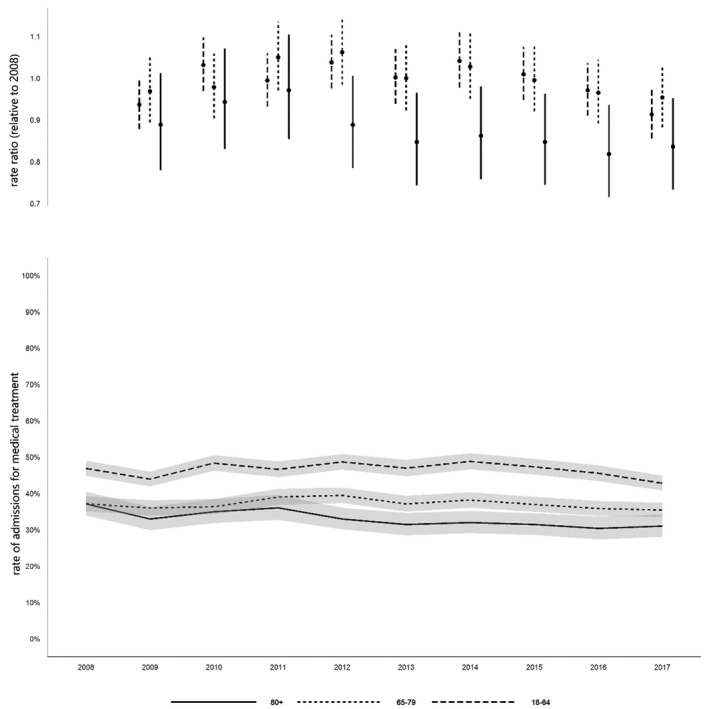
Rates of admission for medical treatment 2008–2017. Values are rate ratios relative to 2008, and rate of admission with medical diagnosis, with 95%CIs.

The youngest age group had the highest proportion of unplanned admissions to critical care (77.2%); the highest rate of planned admissions was seen in the older age group (29.3%; Table 1). The proportions of planned admissions per age cohort associated with surgical diagnoses were significantly greater among older and oldest cohorts from 2015 to 2017, compared with our reference year (2008; Fig 3); unplanned surgical admissions were highest in the oldest cohort, and significantly greater among the oldest cohort from 2012 onwards compared with the reference year. The corresponding decreasing trend in medical admissions over the period was statistically significant among the oldest group (χ^2^ = 27.47, p < 0.001), with an apparent decrease relative to the reference year which predated the introduction of PACUs in 2015 (Fig. 4).

The youngest age group had the highest rate of admissions involving advanced respiratory support overall (invasive mechanical ventilation, 44.9%; Table 1), and significant increases were observed over the study period from 39.6% in 2008 to 44.8% in 2017, a relative increase of 13% (RR 95%CI 1.13 (1.06–1.21)). The rate of admissions involving advanced respiratory support in the older (38.1% overall) and oldest age groups (31.2%) did not change significantly over the study period. The older age group had the highest proportion of admissions involving renal support overall (11.7%), but this decreased significantly over the study period from 13.7% in 2008 to 11.7% in 2017, a relative decrease of 15% (RR 95%CI 0.85 (0.75–0.97)). The oldest age group had the lowest proportion of admissions requiring renal support (8.4%), which did not decrease significantly (χ^2^ = 0.17, *p* = 0.68). The proportion of admissions among the youngest age group requiring renal support was 9.8% overall and did not change over the study period (RR 95%CI 0.98 (0.85–1.12)). The older age group also had the highest proportion of admissions involving advanced cardiovascular support (16.4%), which decreased significantly over the study period from 20.3% in 2008 to 14.2% in 2017, a 30% decrease (RR 95%CI 0.70 (0.62–0.78)). The proportion of admissions in the youngest age group involving advanced cardiovascular support also significantly decreased over the study period from 15.1% in 2008 to 10.8% in 2017, representing a relative decrease of 28% (RR 95%CI 0.72 (0.64–0.81)). The overall proportion of admissions involving advanced cardiovascular support in the oldest age group was 14.7%, which did not change significantly across years (RR 95%CI 0.94 0.77–1.14)).

**Figure 5:**
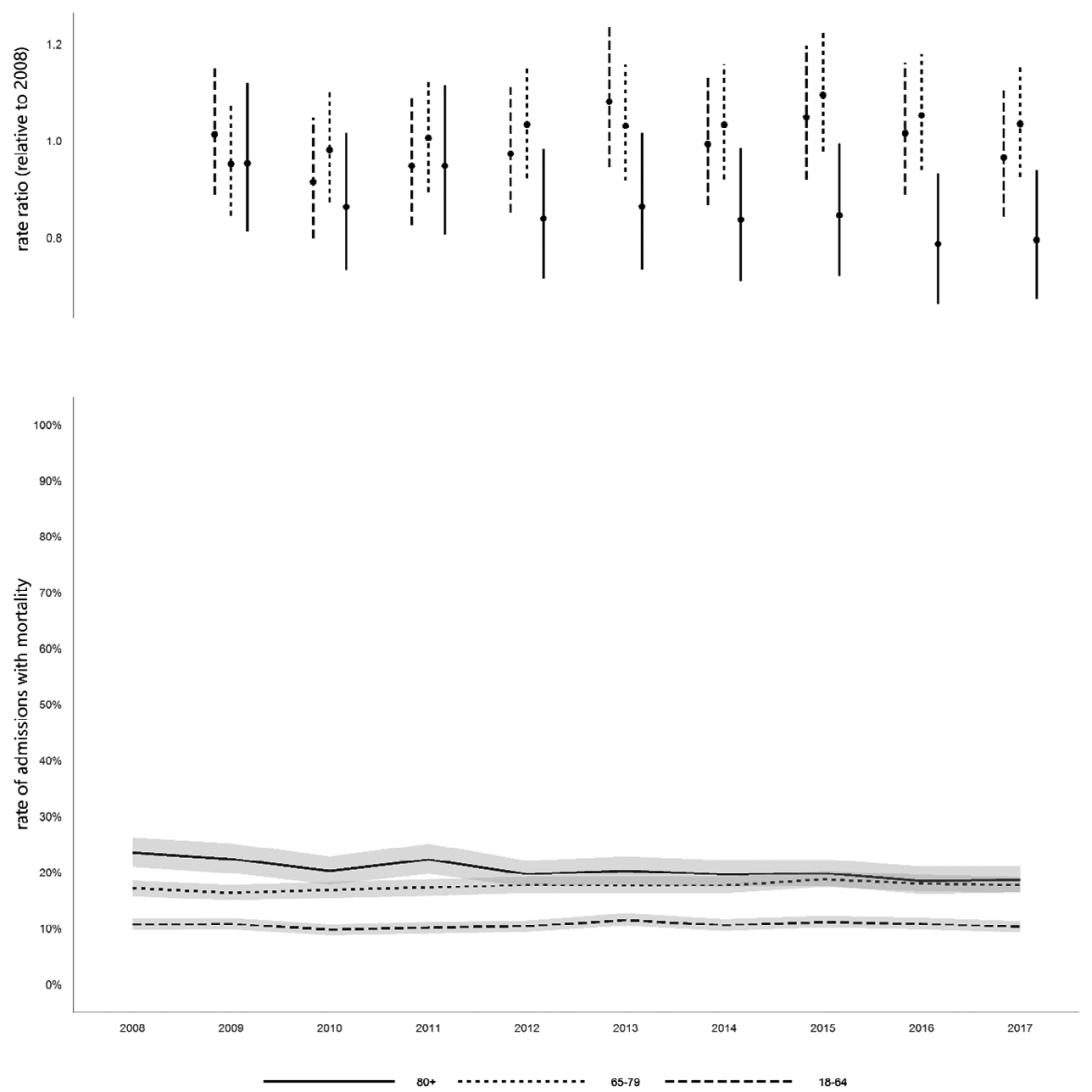
Critical care mortality rate. Values are rate ratio relative to 2008, and rate of admissions with mortality, with 95%CIs.

Critical care and post‐critical care hospital mortality, and one‐year post‐discharge mortality increased with increasing cohort age (online Supporting Information Table S5). Overall hospital mortality and one‐year mortality were 13.8% and 16.1% in the youngest; 24.8% and 30.5% in the older; and 33.3% and 44.2% in the oldest groups, respectively (Table 1). A significant decrease in mortality during critical care was observed with time in the older and oldest groups (Fig. 5), in post‐critical care hospital mortality in all three age groups, and in post‐discharge one‐year mortality in older age groups, but not in the youngest or oldest groups, though numbers were relatively low (online Supporting Information Table S5).

## Discussion

Critical care capacity decreased slightly over the study period, while the national population increased, particularly among those aged > 65 years. There was a significant decrease in overall admissions per 10,000 population, with a 15% relative decrease among the oldest group (≥ 80 years), and a 16% decrease in the older group (65–79 years). There was a decrease in the proportion of admissions with high comorbidity across all age groups, an increase in the proportion of older admissions who were considered ’fit’, and a decrease in the proportion of admissions with a medical diagnosis (particularly among the oldest group). In terms of organ support, we observed an increase in younger patients requiring invasive mechanical ventilation. Lastly, in the short term, there were improvements in critical care and post‐critical care hospital unadjusted mortality among the older and oldest groups and in one‐year mortality among those aged 65–79 years.

Comparisons between large‐scale studies of critical care admission trends are made more challenging by differences in baseline capacity, changes in capacity over time, and national population trends – factors which are not described consistently in the literature. However, our findings contrast with reports of rising trends in proportions of admissions aged ≥ 80 years in: Australia (5.6% increase per year, 2000–2005) [5]; Austria (increase from 11.5 to 15.3%, 1998–2008) [6]; the Republic of Korea (increase from 8.6 to 13.6%, 2002–2010) [23]; and Denmark (increase in from 11.7 to 13.8%, 2005–2011) [7]. Of these, Nielsson et al. reported an increase having occurred despite an unchanged proportion of elderly people in the Danish population, suggesting changes in admission policies (and a lower admission threshold) [7]. Importantly, the results also differ substantially from a recent study utilising data from England, Wales and Northern Ireland [9]. Intensive Care National Audit and Research Centre (ICNARC) investigators reported a marked increase in admissions among those aged > 75 years for the period 1997–2016, to a degree exceeding trends for the corresponding age cohorts in the national population. In England, notably, which made by far the biggest contribution to these data, there was an increase in critical care bed capacity of 35% between 1999 and 2006 [24], and a further increase of 15% between 2008 and 2016 according to other data sources [25, 26].

Our findings more closely resemble trends observed by investigators from Canada [10], the Netherlands [8] and Scotland [11]. From Manitoba, Garland et al. reported an overall decrease in critical care episodes between 1999 and 2007, with a reduction in ICU admission in all age cohorts aged > 50 years, and with faster rates of decline among older age groups [10]. Haas et al. noted an overall increase in critical care admissions in the Netherlands, and therefore viewed a lack of increase in admissions in those aged ≥ 80 years (in contrast to national demographic ageing) between 2005 and 2014 as evidence of changes in admission decision‐making rather than resource limitation [8]. In Scotland, Docherty et al. observed a 22% relative decrease in admissions among population aged ≥ 80 years, and 16% among those aged 65–79 years between 2005 and 2009, which raised concerns with regard to rationing according to chronological age and variation in access to critical care [11].

Critical care capacity in Wales did not increase over the 10‐year study period in line with national demographic changes, but in fact decreased. The decline in admission cohorts aged ≥ 65 years relative to the national population are on a scale similar to that observed in Scotland [11]. We observed a similar preponderance of surgical patients among the oldest cohort to Docherty at al. (approximately two‐thirds), although with a higher proportion of surgical patients among those aged 65–79 years than in Scotland. Importantly, we also observed a significant decreasing trend in medical admissions throughout the study period. Docherty et al. considered that outcomes from surgical conditions are viewed more favourably than medical conditions (this was supported by their observation that among their oldest cohort, one‐year survival was 45% among those undergoing emergency abdominal surgery compared with 25% among those admitted to ICU with pneumonia). During our study period, variation in critical care admission for high‐risk surgical patients is likely to have diminished with the introduction of national quality improvement targets [27, 28, 29]. Indeed, the rise in unplanned surgical admissions among the oldest cohort from 2011 to 2013 appears to coincide with the publication of the Royal College of Surgeons Report on the Peri‐operative Care of the Higher Risk General Surgical Patient [27]. Target‐focused administrative concerns over the progress of elective surgery (vs. harder to measure non‐elective medical demands) and critical care benchmarking processes that at present do not directly account for frailty may have added impetus. Notably, the recent national initiative to improve care of the critically ill in Wales has primarily focused on enhanced care following surgery rather than core critical care capacity [30].

Importantly, our study also adds to the limited literature describing longitudinal trends for comorbidity. We previously reported the predictive value of the modified Charlson comorbidity index [19] in determining long‐term survival following discharge from Welsh critical care units [31]. Applying this same method to a larger, less selected cohort, we observed a prevalence of high comorbidity (modified Charlson comorbidity index > 10) among Welsh patients (63.8%), greater than for Danish (modified Charlson comorbidity index 3 or more, 16.8%) [32] and Scottish patients (three or more comorbidities, 2.8%) [11] to a degree that warrants further investigation. However, examining trends, the significant decrease in proportions of patients with high comorbidity in the study period has not previously been reported, contrasts with data from other parts of the UK [9], and must be considered in the context of capacity constraint. We are unaware of other reports of longitudinal trends in critical care admission according to frailty; we applied the eFI, which was developed and validated in a UK population [20] and implemented in Wales [21]. Using this methodology, the proportion of patients aged ≥ 65 years identified as ‘non‐fit’ (60.6%) was similar to the proportions identified using frailty indices among those aged ≥ 65 years in a Chinese geriatric ICU (60%) [33], and those aged ≥ 16 years in Brazilian ICUs (68.6%) [34]. Among those aged 65–79 years, we found an increased proportion of ‘fit’ patients and decreases in those with mild frailty over time. Further work is required to explore the potential effect of ‘look‐back’ on eFI trends, restricted to those with a complete primary care record for the period under review, but our initial data do not currently support expectations voiced in the literature of “*increased numbers of frail patients being admitted to intensive care units*” [35].

Although not our primary aim, significant improvements in critical care and post‐critical care hospital unadjusted mortality were seen in all age groups with time, and in one‐year mortality among those aged 65–79 years. This is consistent with other large‐scale studies reporting improvements in short‐term [6] and long‐term mortality [36] particularly among older patients, but requires further exploration of the contributions of changing case‐mix and illness severity.

The role of intensive care consultant as gatekeeper is recognised in healthcare systems with high and low numbers of critical care beds, although in the USA (with approximately 30 beds per 100,000 population, see online Supporting Information Table S1) it is a perceived disproportionate ease of access that has led to recent concern [37, 38, 39]. To UK consultants, age and severity of comorbidity were recently reported as the most important patient‐related factors in ICU admission decision‐making [38]. National professional guidance increasingly promotes the importance of patient‐centred care [40], and considerations of the longer‐term impact of critical illness, frailty, age and comorbidity will undoubtedly have played a crucial role in such discussions over the study period. Indeed, the improvements in unadjusted one‐year mortality we observed likely point towards a robust selection process. However, considering the training pathways and professional guidance common to the UK nations, the striking divergence in Wales from ICNARC‐reported admission numbers among older cohorts, those with significant comorbidity and those with a medical diagnosis, seems far more likely a consequence of differences in capacity than primary variance of clinician perspectives and admission thresholds. Faced with increasing competition for a finite resource, it would appear that clinicians have increasingly admitted those considered most likely to benefit and for whom there is a surgical imperative to proceed – but did not include those who may have been admitted (and potentially benefited) in an alternative healthcare system.

The limitations of our study include the absence of illness severity data and detailed diagnostic coding; we were unable to identify PACU patients specifically (although PACU beds only became operational at the very end of the study period). We did not attempt to characterise critical care re‐admissions within the cohort, and this would make for worthwhile further investigation given the perceived increasing competition for resources. Finally, given that this was not the primary aim of our study, we have not attempted to establish trends in adjusted mortality. However, the strengths of our study include the size of population and longitudinal trends, our ability to contextualise on the basis of critical care capacity, and our characterisation of comorbidity and frailty. To the best of our knowledge, this is the first large‐scale study from the UK to report on frailty prevalence among unselected critical care admissions using the eFI.

In conclusion, in contrast to a number of recent reports, we have identified a significant decline in admissions of older patients (aged ≥ 65 years) relative to the national population, of those with comorbidity and those with a medical diagnosis. Multiple factors are likely to have contributed to these trends, but capacity constraint combined with surgical imperative appears to have been important. In comparison with other healthcare systems, we would argue that critical care capacity has failed to keep pace with the needs of an ageing population in Wales.

## Supporting information


**Figure S1**. Critical care admissions by year and per 10,000 population.

**Table S1**. Comparative numbers of critical care beds for UK and other developed nations.

**Table S2**. Critical care capacity in Wales from 2008 to 2017.

**Table S3**. Trends in Critical care admissions.

**Table S4**. Population estimates for Wales.

**Table S5**. Critical care mortality, post‐critical care hospital mortality, and post‐discharge 1‐year mortality.
