# Supplementary material for: Long‐term trends in critical care admissions in Wales *
Source: Anaesthesia. 2021 May 2;76(10):1316–25. doi: 10.1111/anae.15466 (PMC10138728; doi:10.1111/anae.15466)

Supplementary Figure S1. Critical care admissions by year (bar) and per 10,000 population (line). Rate ratios are presented per 10,000 population relative to 2008

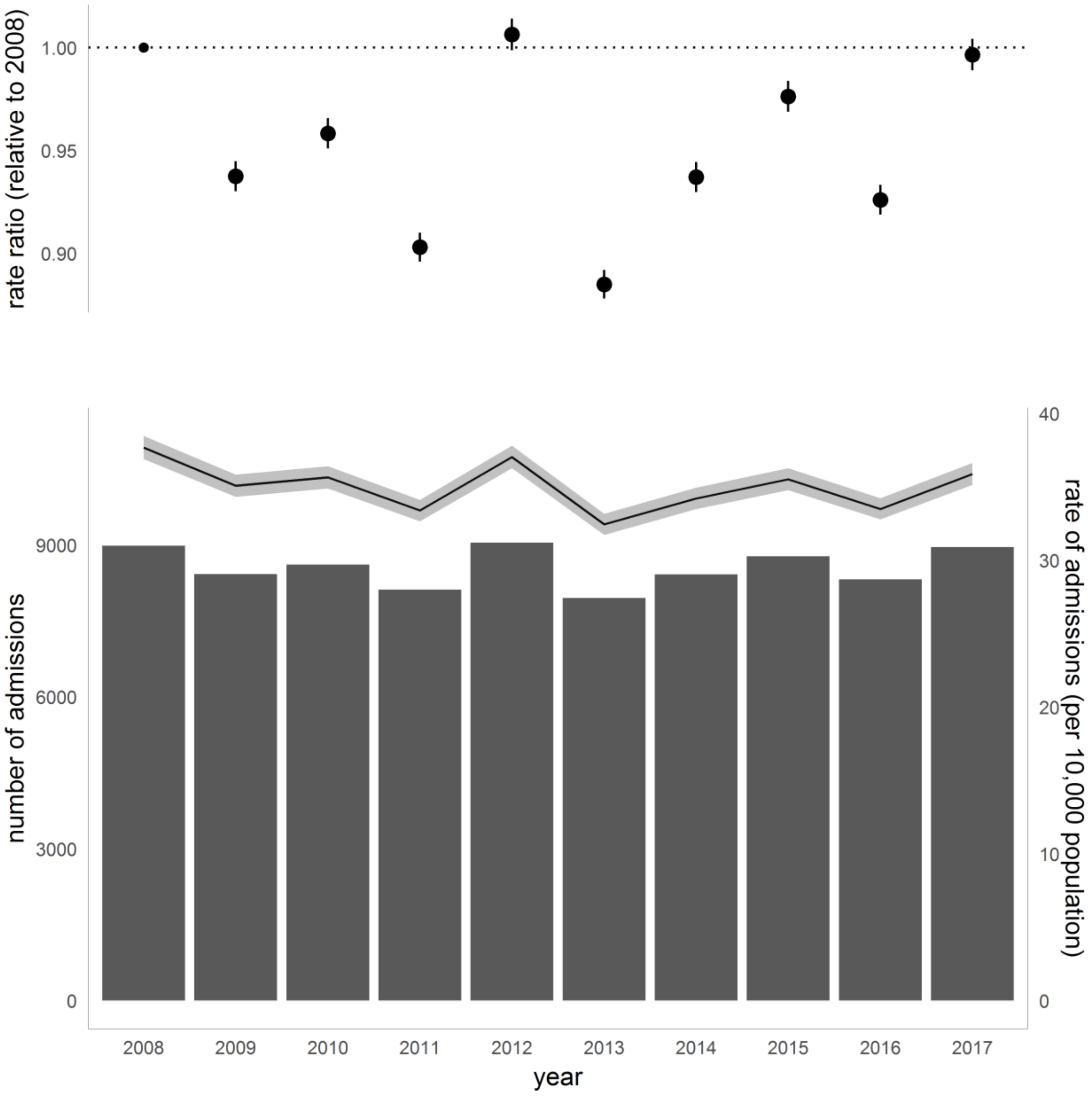

Supplement: Supplementary file 1 — Figure S1. Critical care admissions by year and per 10,000 population. [file ANAE-76-1316-s001.pdf]
