# Supplementary material for: Long‐term trends in critical care admissions in Wales *
Source: Anaesthesia. 2021 May 2;76(10):1316–25. doi: 10.1111/anae.15466 (PMC10138728; doi:10.1111/anae.15466)
Supplement: Supplementary file 2 — Table S1. Comparative numbers of critical care beds for UK and other developed nations. [file ANAE-76-1316-s002.docx]

**Table S1.** Comparative numbers of critical care beds (including paediatric beds) for UK and other developed nations

| **Nation** | **Population**  **(millions)** | **No. critical care (adult/ paediatric) beds** | **No. critical care beds per 100,000 population** | **Reference year** |
| --- | --- | --- | --- | --- |
| England | 56.0 [1] | 3976 [2] | 7.1 | 2017 |
| Scotland | 5.4 [1] | 547 [3-5] | 10.1 | 2016-17 |
| Wales | 3.1 [1] | 186 [5] | 6.0 | 2017 |
| Northern Ireland | 1.9 [1] | 98 [4,5] | 5.2 | 2017 |
| Ireland | 4.8 [6] | 272 [5,7] | 5.7 | 2016-17 |
| Germany | 82.8 [8] | 28,031 [9] | 33.9 | 2017 |
| France | 67.0 [10] | 5671[11] | 8.5 | 2018 |
| Italy | 60.6 [10] | 5184 [11] | 8.6 | 2018 |
| USA | 326.2 [12] | 96,596 [13]* | 29.6 | 2018 |
| Australia | 25.0 [14] | 2229 [14] | 8.9 | 2017 |

* This includes neonatal intensive care beds.

International comparison of adult and paediatric critical care beds. Although attempts have been made to standardise definitions as far as possible, differences persist through variations in reporting metrics; USA figures include neonatal beds, Level 2 “HDU” beds may have been incompletely captured, and private critical care capacity has not been represented for some nations (e.g. England, Ireland).

**References**

1. Stats Wales. National level population estimates by year, age and UK country, 2020. <https://statswales.gov.wales/Catalogue/Population-and-Migration/Population/Estimates/nationallevelpopulationestimates-by-year-age-ukcountry> (accessed 7th December/2020).

2. NHS England. Critical Care Capacity, 2020. <https://www.england.nhs.uk/statistics/statistical-work-areas/critical-care-capacity/> (accessed 7th December/2020).

3. Scottish Intensive Care Society Audit Group. Audit of Critical Care in Scotland 2017: Reporting on 2016, 2017. <https://www.sicsag.scot.nhs.uk/docs/2017/2017-08-08-SICSAG-Report.pdf?55> (accessed 7th December/2020).

4. Faculty of Intensive Care Medicine/ Intensive Care Society. Guidelines for the Provision of Intensive Care Services, Edition 2, 2019. <https://www.ficm.ac.uk/sites/default/files/gpics-v2.pdf> (accessed 7th December/2020).

5. Paediatric Intensive Care Audit Network. Annual Report 2018: Appendices, 2018. <https://www.picanet.org.uk/wp-content/uploads/sites/25/2018/11/PICANet-Annual-Report-Appendices-2018_v1.0.pdf> (accessed 7th December/2020).

6. Central Statistics Office. Census of Population 2016 - Profile 3: An Age Profile of Ireland, 2016. <https://www.cso.ie/en/releasesandpublications/ep/p-cp3oy/cp3/assr/> (accessed 7th December/2020).

7. National Office of Clinical Audit. Irish National ICU Audit Annual Report 2017, 2019. <https://jficmi.anaesthesia.ie/wp-content/uploads/2019/02/Irish_National_ICU_Audit_Annual_Report_2017_FINAL.pdf> (accessed 7th December/2020).

8. Destatis Statistiches Bundesamt. Bevolkerung in Deutschland, 2017. <https://service.destatis.de/bevoelkerungspyramide/index.html#!y=2017&a=18,67&v=2&g> (accessed 7th December/2020).

9. Destatis Statistiches Bundesamt. Gesundheit Grunddaten der Krankenhäuser, 2017, 2018. <https://www.gbe-bund.de/gbe/pkg_isgbe5.prc_isgbe?p_uid=gast&p_aid=90602103&p_sprache=D> (accessed 7th December/2020).

10. Eurostat. News release: EU population 2017, 2018. <https://ec.europa.eu/eurostat/documents/2995521/9063738/3-10072018-BP-EN.pdf/ccdfc838-d909-4fd8-b3f9-db0d65ea457f> (accessed 7th December/2020).

11. Bauer J, Bruggmann D, Klingelhofer D, et al. Access to intensive care in 14 European countries: a spatial analysis of intensive care need and capacity in the light of COVID-19. *Intensive Care Medicine* 2020; **46:** 2026-34.

12. United States Census Bureau. 2018 National and State Population Estimates, 2018. <https://www.census.gov/newsroom/press-kits/2018/pop-estimates-national-state.html> (accessed 7th December/2020).

13. Society of Critical Care Medicine. United States Resource Availability for COVID-19, 2020. <https://www.sccm.org/Blog/March-2020/United-States-Resource-Availability-for-COVID-19> (accessed 7th December/2020).

14. Australian and New Zealand Intensive Care Society. ANZICS Centre for Outcome and Resource Evaluation 2018 Report, 2018. <https://www.anzics.com.au/wp-content/uploads/2019/10/2018-ANZICS-CORE-Report.pdf> (accessed 7th December/2020).
