# Supplementary material for: Long‐term trends in critical care admissions in Wales *
Source: Anaesthesia. 2021 May 2;76(10):1316–25. doi: 10.1111/anae.15466 (PMC10138728; doi:10.1111/anae.15466)
Supplement: Supplementary file 3 — Table S2. Critical care capacity in Wales from 2008 to 2017. [file ANAE-76-1316-s003.docx]

**Table S2** Critical care capacity in Wales: from 2008 to 2017

| **Health Board** | **Hospital** | **Total capacity 2008** | **Total capacity** ‡ **2017** |
| --- | --- | --- | --- |
| Aneurin Bevan University Health Board | Nevill Hall Hospital, Abergavenny | 22 | 29 |
|  | Royal Gwent Hospital, Newport |  |  |
| Abertawe Bro Morgannwg University Health Board * | Morriston Hospital, Swansea | 45 | 36 |
|  | Neath Port Talbot Hospital |  |  |
|  | Princess of Wales Hospital, Bridgend |  |  |
|  | Singleton Hospital, Swansea |  |  |
| Betsi Cadwaladr University Health Board | Wrexham Maelor Hospital | 34 | 34 |
|  | Ysbyty Glan Clwyd, Bodelwyddan |  |  |
|  | Ysbyty Gwynedd, Bangor |  |  |
| Cardiff and Vale University Health Board | Llandough Hospital, Cardiff | 33 | 32 |
|  | University Hospital Wales |  |  |
| Cwm Taf University Health Board † | Prince Charles Hospital, Merthyr Tydfil | 17 | 16 |
|  | Royal Glamorgan Hospital, Llantrisant |  |  |
| Hywel Dda University Health Board | Bronglais General Hospital, Aberystwyth | 27 | 29 |
|  | Glangwili General Hospital, Carmarthen |  |  |
|  | Prince Phillip Hospital,Llanelli |  |  |
|  | Withybush General Hospital, Haverfordwest |  |  |
| Powys Teaching Health Board | n/a |  |  |
| **Total** |  | 178 | 176 |

* Following realignment of the Princess of Wales Hospital, Bridgend, this has now been renamed Swansea Bay University Health Board. † Following realignment of the Princess of Wales Hospital, Bridgend, this has now been renamed Cwm Taf Morgannwg University Health Board. ‡ Includes 9 PACU beds
