# Supplementary material for: Long‐term trends in critical care admissions in Wales *
Source: Anaesthesia. 2021 May 2;76(10):1316–25. doi: 10.1111/anae.15466 (PMC10138728; doi:10.1111/anae.15466)
Supplement: Supplementary file 4 — Table S3. Trends in Critical care admissions. [file ANAE-76-1316-s005.docx]

**Table S3** Trends in critical care admissions. Values are number of admissions to critical care (n), and rate (r) per 10,000 population with 95% CI. p values are presented for chi-square test for trends for rate per 10,000 population.

|  | | Year | | | | | | | | | |  |
| --- | --- | --- | --- | --- | --- | --- | --- | --- | --- | --- | --- | --- |
| Age | | 2008 | 2009 | 2010 | 2011 | 2012 | 2013 | 2014 | 2015 | 2016 | 2017 | p |
| 18-64 | n | 4151 | 4090 | 4117 | 3764 | 4161 | 3581 | 3822 | 3996 | 3792 | 4077 | p=0.34 |
|  | r | 22.5 | 22 | 22.1 | 20.2 | 22.4 | 19.3 | 20.7 | 21.6 | 20.5 | 22 |  |
|  |  | (21.8 -23.2) | (21.4 - 22.7) | (21.5 - 22.8) | (19.5 - 20.8) | (21.7 - 23.1) | (18.7 - 20) | (20 - 21.4) | (21 - 22.3) | (19.8 - 21.2) | (21.3 - 22.7) |  |
| 65-79 | n | 3474 | 3090 | 3213 | 3148 | 3401 | 3066 | 3256 | 3389 | 3313 | 3578 | p<0.001 |
|  | r | 89.2 | 77.8 | 79.4 | 76.5 | 79.4 | 69.4 | 71.9 | 73.4 | 70.7 | 75.3 |  |
|  |  | (86.3 - 92.3) | (75.1 - 80.6) | (76.7 -82.2) | (73.8 - 79.2) | (76.7 - 82.1) | (66.9 -71.9) | (69.4 - 74.4) | (71.0 - 75.9) | (68.3 -73.1) | (72.9 - 77.8) |  |
| 80+ | n | 1364 | 1247 | 1284 | 1205 | 1484 | 1308 | 1345 | 1391 | 1219 | 1303 | p<0.001 |
|  | r | 91.5 | 82.9 | 84.1 | 77.9 | 94.3 | 82.4 | 83.2 | 85.2 | 73.5 | 77.5 |  |
|  |  | (86.7 -96.4) | (78.3 - 87.6) | (79.6, -88.8) | (73.5 -82.4) | (89.6 -99.3) | (78.0 – 87.0) | (78.8 - 87.8) | (80.8 - 89.8) | (69.4 - 77.7) | (73.3 -81.8) |  |
