# Supplementary material for: Long‐term trends in critical care admissions in Wales *
Source: Anaesthesia. 2021 May 2;76(10):1316–25. doi: 10.1111/anae.15466 (PMC10138728; doi:10.1111/anae.15466)
Supplement: Supplementary file 5 — Table S4. Population estimates for Wales. [file ANAE-76-1316-s006.docx]

**Table S4** Population estimates for Wales. Values are number (proportion).

|  | Year | | | | | | | | | |
| --- | --- | --- | --- | --- | --- | --- | --- | --- | --- | --- |
| Age | 2008 | 2009 | 2010 | 2011 | 2012 | 2013 | 2014 | 2015 | 2016 | 2017 |
| 18 to 64 | 1,847,571 | 1,855,000 | 1,859,612 | 1,864,932 | 1,857,283 | 1,851,571 | 1,847,680 | 1,846,425 | 1,850,607 | 1,853,607 |
|  | 77.4% | 77.2% | 76.9% | 76.7% | 76.0% | 75.5% | 75.0% | 74.7% | 74.5% | 74.2% |
| 65 to 79 | 389,257 | 397,144 | 404,586 | 411,619 | 428,558 | 441,895 | 453,057 | 461,538 | 468,776 | 475,079 |
|  | 16.3% | 16.5% | 16.7% | 16.9% | 17.5% | 18.0% | 18.4% | 18.7% | 18.9% | 19.0% |
| 80 | 149,144 | 150,453 | 152,669 | 154,774 | 157,320 | 158,735 | 161,690 | 163,235 | 165,861 | 168,190 |
|  | 6.3% | 6.3% | 6.3% | 6.4% | 6.4% | 6.5% | 6.6% | 6.6% | 6.7% | 6.7% |
| All | 2,385,972 | 2,402,597 | 2,416,867 | 2,431,325 | 2,443,161 | 2,452,201 | 2,462,427 | 2,471,198 | 2,485,244 | 2,496,876 |

National population mid-year estimates for Wales by year.
