# Supplementary material for: Long‐term trends in critical care admissions in Wales *
Source: Anaesthesia. 2021 May 2;76(10):1316–25. doi: 10.1111/anae.15466 (PMC10138728; doi:10.1111/anae.15466)
Supplement: Supplementary file 6 — Table S5. Critical care mortality, post‐critical care hospital mortality, and post‐discharge 1‐year mortality. [file ANAE-76-1316-s004.docx]

**Table S5** Critical care (ICU) mortality, post-critical care hospital (Hospital) mortality, and post-discharge 1-year (1-year) mortality. Values are number (proportion).

|  | | Year | | | | | | | | | |  |
| --- | --- | --- | --- | --- | --- | --- | --- | --- | --- | --- | --- | --- |
| Age | | 2008 | 2009 | 2010 | 2011 | 2012 | 2013 | 2014 | 2015 | 2016 | 2017 | P value |
| 18-64 | n | 4,151 | 4,090 | 4,117 | 3,764 | 4,161 | 3,581 | 3,822 | 3,996 | 3,792 | 4,077 |  |
|  | ICU | 441 | 440 | 400 | 379 | 430 | 411 | 403 | 445 | 409 | 418 | 0.47 |
|  |  | 10.6% | 10.8% | 9.7% | 10.1% | 10.3% | 11.5% | 10.5% | 11.1% | 10.8% | 10.3% |  |
|  | Hospital | 146 | 147 | 165 | 113 | 145 | 127 | 120 | 126 | 108 | 93 | <0.01 |
|  |  | 3.5% | 3.6% | 4.0% | 3.0% | 3.5% | 3.5% | 3.1% | 3.2% | 2.8% | 2.3% |  |
|  | n* | 3,663 | 3,382 | 3,335 | 2,970 | 3,264 | 2,776 | 2,932 | 3,132 | 2,980 | 3,137 |  |
|  | 1-year | 120 | 116 | 116 | 89 | 101 | 89 | 77 | 88 | 69 | 58 | 0.21 |
|  |  | 3.3% | 3.4% | 3.5% | 3.0% | 3.1% | 3.2% | 2.6% | 2.8% | 2.3% | 1.8% |  |
| 65-79 | n | 3,474 | 3,090 | 3,213 | 3,148 | 3,401 | 3,066 | 3,256 | 3,389 | 3,313 | 3,578 |  |
|  | ICU | 595 | 504 | 540 | 542 | 602 | 541 | 576 | 635 | 597 | 634 | 0.02 |
|  |  | 17.1% | 16.3% | 16.8% | 17.2% | 17.7% | 17.6% | 17.7% | 18.7% | 18.0% | 17.7% |  |
|  | Hospital | 273 | 260 | 261 | 261 | 254 | 215 | 238 | 236 | 186 | 224 | <0.01 |
|  |  | 7.9% | 8.4% | 8.1% | 8.3% | 7.5% | 7.0% | 7.3% | 7.0% | 5.6% | 6.3% |  |
|  | n* | 3,072 | 2,625 | 2,747 | 2,570 | 2,775 | 2,486 | 2,654 | 2,761 | 2,715 | 2,952 |  |
|  | 1-year | 224 | 208 | 207 | 199 | 186 | 167 | 179 | 191 | 142 | 181 | <0.01 |
|  |  | 7.3% | 7.9% | 7.5% | 7.7% | 6.7% | 6.7% | 6.7% | 6.9% | 5.2% | 6.1% |  |
| 80+ | n | 1,364 | 1,247 | 1,284 | 1,205 | 1,484 | 1,308 | 1,345 | 1,391 | 1,219 | 1,303 |  |
|  | ICU | 320 | 279 | 260 | 268 | 292 | 265 | 264 | 276 | 225 | 243 | <0.01 |
|  |  | 23.5% | 22.4% | 20.2% | 22.2% | 19.7% | 20.3% | 19.6% | 19.8% | 18.5% | 18.6% |  |
|  | Hospital | 180 | 191 | 172 | 186 | 200 | 174 | 157 | 162 | 124 | 144 | <0.01 |
|  |  | 13.2% | 15.3% | 13.4% | 15.4% | 13.5% | 13.3% | 11.7% | 11.6% | 10.2% | 11.1% |  |
|  | n* | 1,241 | 1,133 | 1,158 | 1,062 | 1,274 | 1,128 | 1,185 | 1,207 | 1,034 | 1,119 |  |
|  | 1-year | 158 | 167 | 147 | 160 | 174 | 142 | 131 | 137 | 103 | 122 | 0.24 |
|  |  | 12.7% | 14.7% | 12.7% | 15.1% | 13.7% | 12.6% | 11.1% | 11.4% | 10.0% | 10.9% |  |

* Denominator for 1-year post-hospital discharge mortality is considered for the index critical care admission only. p values are presented for chi-square test for trends.
